# Response to “Are fluids resuscitation the “Keyser Soze” of acute kidney injury in trauma patients?”

**DOI:** 10.1186/s13054-019-2344-6

**Published:** 2019-02-19

**Authors:** Anatole Harrois, Benjamin Soyer, Tobias Gauss, Sophie Hamada, Mathieu Raux, Jacques Duranteau, for the Traumabase® Group, Catherine Paugam-Burtz, Romain Pirracchio, Anne Godier, Sylvain Ausset, Eric Meaudre, Thomas Geeraerts, Nathalie Delhaye, Bernard Vigué

**Affiliations:** 1 Department of Anesthesiology and Critical Care, Bicêtre Hôpitaux Universitaires Paris Sud, Université Paris Saclay, AP-HP, 78 Rue du Général Leclerc, 94275 Le Kremlin Bicêtre, France; 2 Department of Anesthesiology and Critical Care, AP-HP, Beaujon, Hôpitaux Universitaires Paris Nord Val de Seine, 100 Avenue du Général Leclerc, 92110 Clichy, France; 3 Department of Anesthesiology and Critical Care, INSERM, UMRS1158 Neurophysiologie Respiratoire Expérimentale et Clinique, Groupe Hospitalier Pitié-Salpêtrière Charles Foix, Sorbonne Université, AP-HP, 47-83 Boulevard de l’Hôpital, 75013 Paris, France

We appreciate the interest expressed by Dr. Jamme and Ben Hadj Salem in our study recently published in critical care reporting the prevalence and the risk factors of acute kidney injury (AKI) in a multicentre cohort of 3111 trauma patients [1]. The issue they raised was the lack of data on fluid resuscitation, especially regarding the amount of crystalloids and colloids. We share with the authors the view that the choice of fluid is a critical issue to prevent AKI in trauma patients. Indeed, recent studies have called into question the safety of colloids [2] in trauma patients as well as the safety of NaCl 0.9% in ICU patients [3]. Our database provides data on prehospital fluid resuscitation for the whole study cohort as well as data on 24-h fluid resuscitation for hemorrhagic shock patients (*n* = 355, 11%).

Nineteen percent of the 3111 study patients received colloids (median volume of 500 mL [IQR 500–750]) while 94% of the 3111 study patients received crystalloids (median volume of 500 mL [IQR 500–1000]) during the prehospital period. When the volume of colloids or crystalloids administered in the prehospital setting was forced into the predictive model of AKI (all stages of RIFLE classification), odd ratios were respectively (per 1000 mL of solution) 1.34 (CI 0.85–2.12, *p* = 0.21) and 1.11 (CI 0.84–1.45, *p* = 0.47). Thus, prehospital fluid resuscitation does not provide additional value to early predict AKI. Longer period of exposure might be worth considering to capture the potential nephrotoxic effect of fluid on renal function; however, as our main objective was to early predict AKI after trauma, we believe it would make the model less relevant.

Eighty-seven percent of hemorrhagic shock patients received colloids over the first 24 h of care (median volume of 1000 mL [IQR 500–2000]). Though only 9.6% of them received a dose higher than 33 mL/kg, we cannot rule out that colloids caused renal toxicity in our study cohort. In the meantime, patients received a median volume of 3500 mL [IQR 2000–6000] of cristalloids. Our database does not distinguish the various types of cristalloids and provides no data on hyperchloremia to indirectly assess the administered volume of NaCl 0.9%. However, at the time of the study, the three centres were using, though not exclusively, NaCl 0.9% for fluid resuscitation. Given the large amount of administered crystalloids in hemorrhagic shock patients, we cannot exclude that chloride-rich fluids worsened renal aggression.

**Fig. 1:**
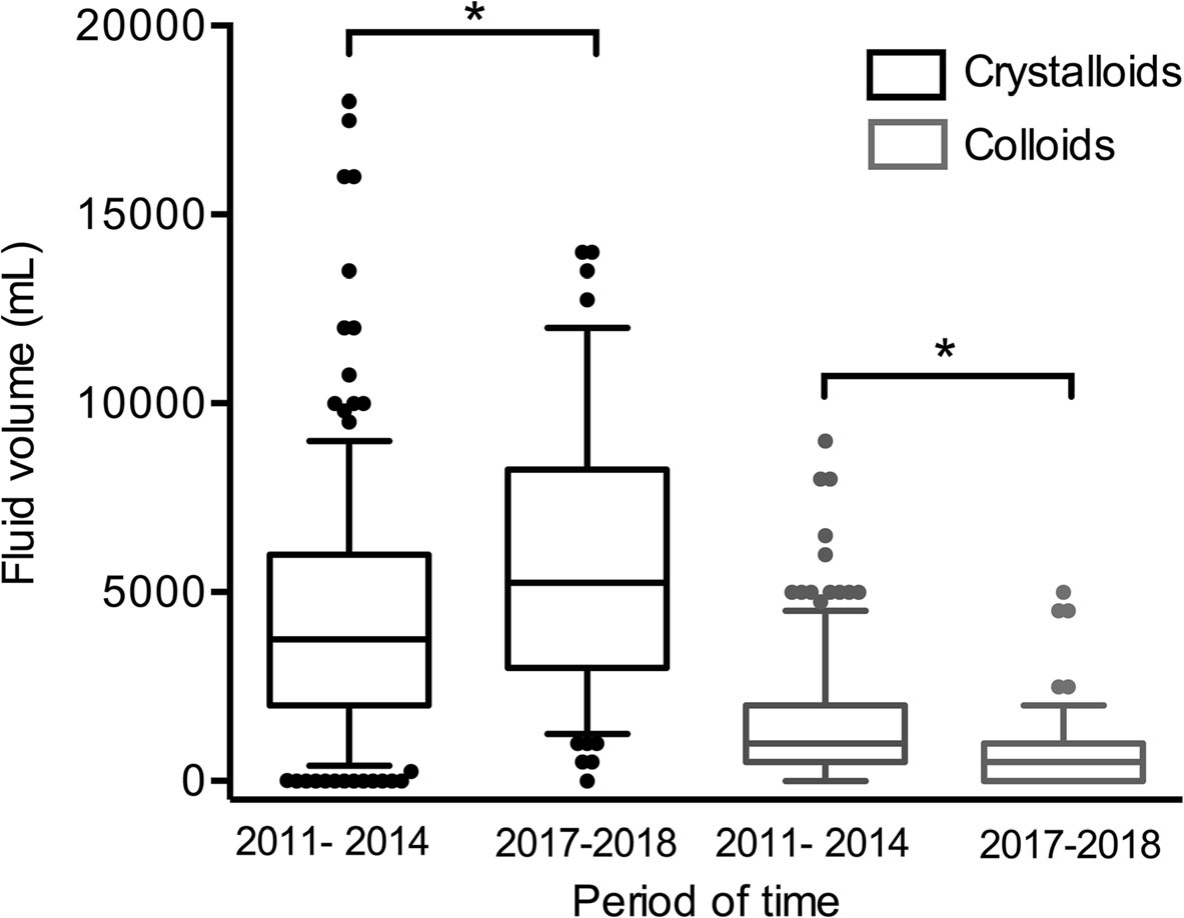
Amounts of colloids and crystalloids administered during the first 24 h in patients with hemorrhagic shock from the study cohort [1] (2011–2014, *n* = 355) and in 129 patients with hemorrhagic shock managed in the same 3 trauma centres over 14 months in 2017–2018. Hemorrhagic shock is defined by the transfusion of at least 4 units of packed red blood cells within the first 6 h. Boxes are represented as median, interquartile range (upper and lower box limit) and 5–95% percentile. *p < 0.001 (Mann-Whitney test)

As shown in Fig. 1, colloid use has declined over the last few years while crystalloids remain the cornerstone for fluid resuscitation in trauma patients. In order to prevent nephrotoxicity related to NaCl 0.9%, isotonic-balanced crystalloids have been proposed for fluid resuscitation, including for patients with traumatic brain injury [4]. However, further studies are necessary to assess if this practice translates to better outcome in trauma patients. We are about to start such a study (NCT03630224).
